# Cushingoid Features and Addisonian Crisis Following Abrupt Withdrawal of Potent Topical Corticosteroids: A Case Report

**DOI:** 10.7759/cureus.97749

**Published:** 2025-11-25

**Authors:** Shanu Shanavas Khan, Ramesh Yadav, Nirmeen Maali, Vinod Warrier, Aayat Almezel

**Affiliations:** 1 Acute Medicine, Mid and South Essex NHS Foundation Trust, Southend-on-Sea, GBR; 2 Acute Medicine, Southend University Hospital, Southend-on-Sea, GBR; 3 Internal Medicine, Mid and South Essex NHS Foundation Trust, Southend-on-Sea, GBR

**Keywords:** addisonian crisis, adrenal insufficiency, iatrogenic cushing’s syndrome, psoriasis, septic shock, steroid withdrawal, topical corticosteroids

## Abstract

We report the case of a 58-year-old man with psoriasis who developed Addisonian crisis and septic shock following abrupt cessation of long-term, unsupervised use of potent topical corticosteroids, obtained without a prescription. He presented with hypotensive septic shock due to community-acquired pneumonia, compounded by adrenal suppression from chronic clobetasol and betamethasone dipropionate use. A short Synacthen test confirmed adrenal insufficiency (basal cortisol 25 nmol/L, 30-minute value 40 nmol/L). Management included aggressive intravenous fluids, vasopressor support, broad-spectrum antibiotics, and intravenous hydrocortisone, resulting in progressive recovery.

This case highlights the systemic risks associated with unsupervised use of high-potency topical corticosteroids. Chronic misuse can lead to Cushingoid features, proximal muscle weakness, features of metabolic syndrome, and life-threatening adrenal insufficiency following abrupt withdrawal. Clinicians should maintain a high index of suspicion for adrenal crisis in any patient presenting with unexplained hypotension, particularly if they have a Cushingoid appearance, recent steroid cessation, or an intercurrent illness. Improving patient education, ensuring supervised tapering, and tightening the regulation of potent topical corticosteroids are essential to prevent such outcomes.

## Introduction

Cushing’s syndrome refers to the constellation of signs and symptoms caused by chronic exposure to excess glucocorticoids, of endogenous or exogenous origin [1]. Iatrogenic Cushing’s syndrome is the most common form and typically arises from prolonged corticosteroid therapy [2]. Topical corticosteroids remain a cornerstone in the management of psoriasis and other chronic inflammatory dermatoses; however, potent agents such as clobetasol propionate can be absorbed systemically in sufficient quantities to suppress the hypothalamic-pituitary-adrenal (HPA) axis, particularly with extensive application, use on inflamed or thin skin, barrier disruption, occlusion, and prolonged duration [2,3]. Systemic absorption has been quantified in several studies, with as little as 2 g/day of clobetasol for one week shown to suppress cortisol production, and absorption increases up to 10-fold under occlusion [2,3].

Abrupt cessation of corticosteroids can precipitate Addisonian crisis, characterised by hypotension, electrolyte abnormalities, hypoglycaemia, and circulatory collapse [4]. This risk is heightened during intercurrent illness or physiological stress, when cortisol requirements increase [4,5].

We present a case of adrenal crisis and septic shock following abrupt withdrawal of long-term potent topical corticosteroids, emphasising the systemic complications of topical therapies, the need for supervised tapering, and clinician vigilance.

## Case presentation

A 58-year-old man presented with a seven-day history of fever, coryza, productive purulent cough, and malaise. Over the preceding 24 hours, he developed worsening dyspnoea and acute deterioration. His past medical history included hypertension, treated with amlodipine, and type 2 diabetes mellitus, previously managed with metformin and gliclazide; both agents were discontinued one week before admission. He had long-standing psoriasis, managed with multiple super-potent topical corticosteroids (clobetasol propionate, betamethasone dipropionate, fluocinolone acetonide, and mometasone furoate) obtained abroad and used for years without medical supervision [2,3]. Psoriasis management is typically guided by established clinical guidelines [6]. The patient denied using any alternative, herbal, or traditional medicinal preparations for psoriasis, and there was no evidence of adulterated oral or topical formulations beyond the listed potent topical corticosteroids. Importantly, he stopped all topical steroids abruptly one week prior, immediately before developing pneumonia, an important combination that makes this case clinically unique due to the coexistence of prolonged potent steroid misuse, abrupt withdrawal, and acute sepsis as a precipitant for adrenal crisis [7].

On examination, he was acutely unwell but alert and oriented, with a Glasgow Coma Scale of 15/15. He was hypotensive (67/47 mmHg), tachycardic (134 bpm), tachypnoeic (34 breaths/min), hypoxic (89% on room air), and had a temperature of 38°C. Chest auscultation revealed bronchial breathing and coarse crepitations, predominantly over the left lung field. Cardiovascular and neurological examinations were unremarkable.

**Figure 1:**
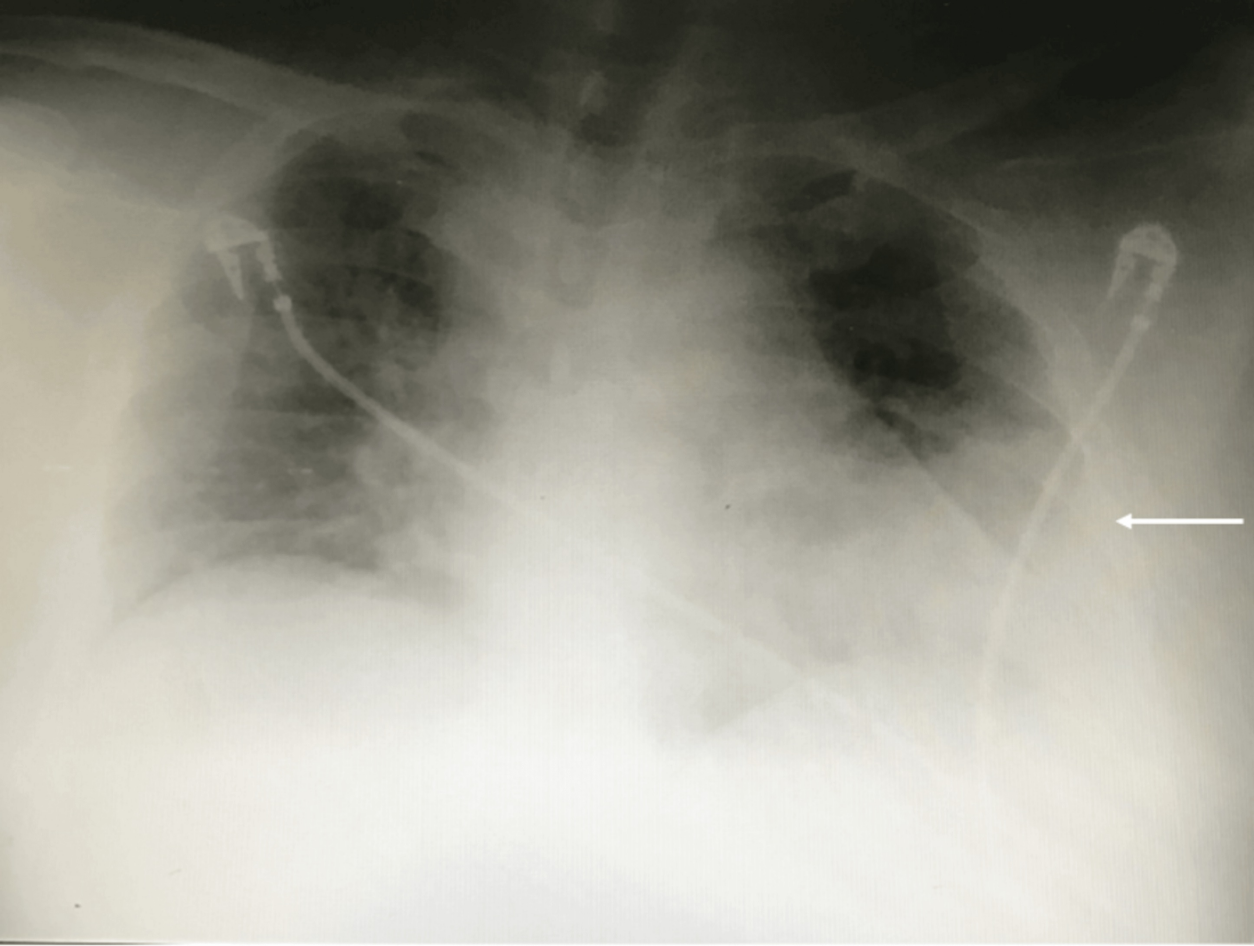
Chest radiograph showing left-sided consolidation (arrow)

A chest radiograph demonstrated extensive left-sided consolidation (Figure 1), consistent with community-acquired pneumonia [8].

**Figure 2:**
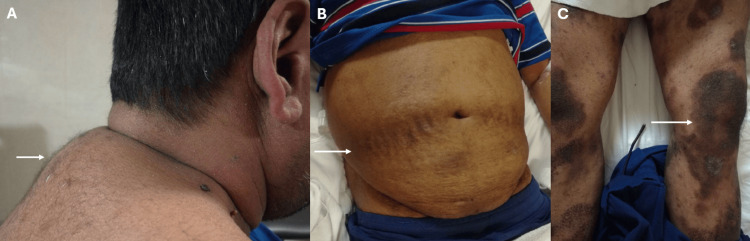
Physical examination findings consistent with Cushingoid features and psoriasis Dorsocervical fat pad consistent with Cushingoid features (arrow) (A); Abdominal striae (arrow) (B); Hyperpigmented psoriatic plaques affecting the lower limbs (arrow) (C).

On further physical examination, he was noted to have Cushingoid features, including a dorsocervical fat pad, wide abdominal striae, and chronic psoriatic plaques over both lower limbs (Figures 2A-2C).

Initial laboratory investigations showed leukocytosis, acute kidney injury, hyponatraemia, hyperkalaemia, and elevated inflammatory markers. Due to clinical suspicion, endocrine testing was performed; he had a random cortisol of 106 nmol/L (consistent with Critical Illness-Related Cortisol Insufficiency (CIRCI)), a basal cortisol of 25 nmol/L, and a 30-minute Synacthen cortisol of 40 nmol/L. These results revealed profoundly suppressed basal cortisol and an inadequate response to a short Synacthen test, confirming adrenal insufficiency [4,5]. All endocrine samples were drawn before any hydrocortisone administration (Table 1).

**Table 1:** Laboratory investigations on admission

Test	Result	Reference Range
Haemoglobin	171 g/dL	130-180 g/dL
WBC	33.6 × 10⁹/L	4.0-11.0 × 10⁹/L
Sodium	133 mmol/L	133-146 mmol/L
Potassium	5.2 mmol/L	3.5-5.3 mmol/L
Urea	7.1 mmol/L	2.5-7.8 mmol/L
Creatinine	212 µmol/L	60-110 µmol/L
Random Glucose	7.7 mmol/L	3.9-7.8 mmol/L
CRP	77 mg/L	<5 mg/L
ESR	45 mm/h	0-20 mm/h
Serum Cortisol (random)	106 nmol/L	185-624 nmol/L
Basal Cortisol (0 min)	25 nmol/L	>185 nmol/L
30-Min Post-synacthen	40 nmol/L	>450 nmol/L

The patient was diagnosed with septic shock secondary to community-acquired pneumonia, compounded by an Addisonian crisis due to abrupt corticosteroid withdrawal.

He received 10 L of intravenous normal saline over 48 hours, with persistent hypotension and oliguria necessitating norepinephrine infusion. Intravenous hydrocortisone 100 mg was given as a bolus, followed by 50 mg every six hours as stress-dose replacement. Once haemodynamically stable, the hydrocortisone was tapered over the following days and transitioned to oral hydrocortisone for maintenance therapy, under endocrinology guidance. Empirical broad-spectrum antibiotics were commenced to address the presumed infectious trigger.

Over subsequent days, he demonstrated progressive recovery, with haemodynamic stabilisation, improved urine output, and resolution of hypoxaemia. He was discharged with endocrinology follow-up for ongoing assessment of adrenal insufficiency and dermatology review for structured psoriasis management. Discharge counselling covered supervised steroid tapering, sick-day rules (dose escalation during febrile illness), and a recommendation to wear a medical alert bracelet identifying adrenal insufficiency.

## Discussion

This case highlights the systemic consequences of chronic, unsupervised use of potent topical corticosteroids. Systemic absorption of clobetasol propionate, a super-potent (Class I) preparation, varies by anatomic site (highest across mucosa and scrotum; lowest over palms and soles) and is influenced by multiple factors, including skin inflammation or barrier disruption (such as fissures), occlusion, potency, duration of use, and total surface area treated [2,3]. In conditions such as psoriasis, where inflamed, fissured, or compromised skin is common, percutaneous absorption is markedly increased [6]. Under such conditions, long-term use can result in significant HPA-axis suppression and Cushingoid features [2,3].

Chronic HPA suppression predisposes patients to adrenal insufficiency. Abrupt corticosteroid withdrawal - especially during physiological stress, such as infection - can precipitate an Addisonian crisis, characterised by refractory hypotension, electrolyte disturbances, and circulatory collapse [3,4,7]. The incidence of adrenal crisis is estimated at 6-8 events per 100 patient-years, with mortality remaining substantial [5,9]. In this patient, community-acquired pneumonia likely increased cortisol demand, unmasking severe adrenal suppression and precipitating shock. Prompt recognition and treatment with stress-dose hydrocortisone, fluid resuscitation, and vasopressor support were life-saving [5,8,10].

Septic shock and adrenal crisis share overlapping features - hypotension, electrolyte abnormalities, and altered mentation - making differentiation challenging. In this case, pneumonia increased cortisol demand, unmasking profound adrenal suppression. The random cortisol value met criteria for CIRCI, but the very low basal cortisol and phenotype supported chronic exogenous suppression.

A random cortisol level below 276 nmol/L is consistent with CIRCI. In this case, the Short Synacthen Test was performed during acute illness. Although repeating the test after recovery would be ideal, the results obtained were still highly suggestive of adrenal dysfunction. The profoundly low basal cortisol, minimal response to Synacthen, and the patient’s marked Cushingoid features from long-term misuse of potent topical corticosteroids strongly supported chronic HPA-axis suppression. Abrupt steroid withdrawal in the setting of acute sepsis likely precipitated an adrenal crisis. Follow-up Synacthen testing after clinical recovery would still be required to confirm ongoing adrenal insufficiency.

Overall, both CIRCI and chronic HPA-axis suppression likely contributed to the patient’s presentation. However, the patient’s extensive history of unsupervised use of super-potent topical corticosteroids, coupled with their clear Cushingoid appearance, strongly suggested underlying chronic adrenal suppression. In this context, sudden cessation of topical steroids, combined with the physiological stress of pneumonia, likely triggered an adrenal crisis, with superimposed CIRCI further exacerbating the severity of shock.

Patients requiring long-term potent topical corticosteroids should undergo periodic endocrine monitoring, such as cortisol assessment or Synacthen testing, to detect HPA-axis suppression early [5,6]. Equally important are patient education regarding the risks of abrupt discontinuation, supervised tapering, and awareness of adrenal crisis symptoms [5,11]. Primary care clinicians play a key role in recognising extensive or atypical skin lesions and in identifying inappropriate corticosteroid use early, ensuring timely referral to dermatology or endocrinology for structured management. At the public health level, unrestricted over-the-counter access to very potent topical corticosteroids in some countries facilitates misuse and delays recognition of systemic toxicity, underscoring the need for regulatory safeguards [10]. Global misuse of potent topical steroids is rising, particularly in South Asia, the Middle East, and Africa, where over-the-counter access is widespread. WHO and dermatology societies warn against unregulated availability and advocate for stricter controls.

## Conclusions

This case underscores the serious systemic consequences of chronic, unsupervised use of super-potent topical corticosteroids, particularly when applied over inflamed or fissured skin, where absorption is significantly enhanced. Long-term exposure led to profound HPA-axis suppression and Cushingoid features, while abrupt discontinuation during acute illness triggered adrenal crisis, further complicated by septic shock and CIRCI. The overlap between septic shock and adrenal insufficiency, presenting with hypotension, electrolyte abnormalities, and altered mentation, highlights the diagnostic challenge and the importance of maintaining a high index of suspicion in similar clinical scenarios. Early recognition and prompt administration of stress-dose corticosteroids remain essential to prevent circulatory collapse and reduce mortality.

Clinicians should suspect adrenal insufficiency in patients presenting with shock, or otherwise unexplained hypotension following steroid use or withdrawal, and initiate stress-dose steroids promptly. Primary care physicians play a key role in identifying extensive, widespread, or atypical skin lesions early and ensuring timely referral to dermatology or other relevant specialties, thereby preventing corticosteroid misuse and enabling appropriate management of psoriasis with supervised steroid tapering. Broader public health measures are urgently needed, particularly in regions where potent topical steroids remain readily accessible over the counter. Strengthened regulatory controls, increased patient education, and coordinated dermatology-endocrinology follow-up are key strategies to prevent misuse, minimise systemic toxicity, and safeguard patient safety.
